# *Clostridioides difficile* Infection in an Italian Tertiary Care University Hospital: A Retrospective Analysis

**DOI:** 10.3390/antibiotics12050837

**Published:** 2023-04-30

**Authors:** Alice Annalisa Medaglia, Alessandro Mancuso, Chiara Albano, Giuseppe Zinna, Luca Pipitò, Cinzia Calà, Rita Immordino, Raffaella Rubino, Silvia Bonura, Baldassare Canino, Giuseppe Calamusa, Claudia Colomba, Pier Luigi Almasio, Antonio Cascio

**Affiliations:** 1 Infectious and Tropical Diseases Unit, AOU Policlinico “P. Giaccone”, 90127 Palermo, Italyantonio.cascio03@unipa.it (A.C.); 2 Antimicrobial Stewardship Team, AOU Policlinico “P. Giaccone”, 90127 Palermo, Italy; giuseppe.calamusa@unipa.it; 3 Department of Health Promotion, Mother and Child Care, Internal Medicine and Medical Specialties, 90127 Palermo, Italy; 4 Microbiology and Virology Unit, AOU Policlinico “P. Giaccone”, 90127 Palermo, Italy

**Keywords:** *Clostridioides difficile* infection, fidaxomicin, vancomycin, metronidazole, bezlotoxumab, hemodialysis

## Abstract

*Clostridioides difficile* infection (CDI) is a significant cause of morbidity and mortality, mostly in frail patients. Notification is not mandatory in Italy, and data on incidence, risk of death, and recurrence are lacking. The purpose of this study was to determine CDI incidence and risk factors for mortality and recurrence. The “ICD-9 00845” code in hospital-standardized discharged forms (H-SDF) and microbiology datasets were used to retrieve CDI cases at Policlinico Hospital, Palermo between 2013 and 2022. Incidence, ward distribution, recurrence rate, mortality, and coding rate were considered. The risk of death and recurrence was predicted through multivariable analysis. There were 275 CDIs, 75% hospital-acquired, the median time between admission and diagnosis was 13 days, and the median stay was 21 days. Incidence increased from 0.3 to 5.6% (an 18.7-fold increase) throughout the decade. Only 48.1% of cases were coded in H-SDF. The rate of severe/severe-complicated cases increased 1.9 times. Fidaxomicin was used in 17.1% and 24.7% of cases overall and since 2019. Overall and attributable mortalities were 11.3% and 4.7%, respectively. Median time between diagnosis and death was 11 days, and recurrence rate was 4%. Bezlotoxumab was administered in 64% of recurrences. Multivariable analysis revealed that only hemodialysis was associated with mortality. No statistically significant association in predicting recurrence risk emerged. We advocate for CDI notification to become mandatory and recommend coding CDI diagnosis in H-SDF to aid in infection rate monitoring. Maximum attention should be paid to preventing people on hemodialysis from getting CDI.

## 1. Introduction

*Clostridioides difficile* is the leading cause of hospital-acquired diarrhea and is often associated with previous antibiotic use. *C. difficile* infection (CDI) represents an emerging infectious disease worldwide, as its incidence and severity are increasing [1,2], significantly impacting morbidity, mortality, and financial cost [3].

Age ≥ 65 years, prior hospitalization, treatment with proton pump inhibitors (PPIs), and antibiotic exposure are known to be the most critical risk factors for developing CDI [4].

In the US and Northern America, CDI rates have been monitored through an active surveillance system since 2000; *C. difficile* is known to be the most common cause of healthcare-associated infections, accounting for 15% of them [5]. Europe still lacks a coordinated CDI surveillance system, but two ECDC-funded surveys, performed in 2005 and 2008, documented a mean CDI incidence of 2.45 and 4.1 per 10,000 patient days (PD) per hospital [2] A European multicentric prospective point prevalence study (PPS) of CDI in hospitalized patients with diarrhea (EUCLID) found an estimated average incidence of 7 per 10,000 PD (range 0.7–28.7) between 2011 and 2013 [6]. In Italy, a multicentric Italian study documented an incidence of 2.3 per 10,000 PD between 2006 and 2011 [7], and the PPS-EUCLID as mentioned above estimated it as 9.5 and 7.2 per 10,000 PD, respectively, in 2011–2012 and 2012–2013 [6].

Also, CDI-associated mortality is reported to have risen from 2% before 2000 to 5 and 7–17%, respectively, in endemic and epidemic settings after 2000 [8].

Few data on severity can be retrieved from the scientific literature as the definition recently changed [9,10]. Greater age and multiple comorbidities are prognostic factors for severe CDI [11].

Severe CDI (sCDI) is characterized by one of the following factors at presentation: body temperature >38.5 °C, leucocyte count >15,000/L, and rise in creatinine (>50% above the baseline). Additional supporting factors are distension of the large intestine, pericolic fat stranding, or colonic wall thickening at imaging [9]. 

Several scores have also been utilized to classify patients with CDI. A Zar score was used to predict the severity of CDI. It considers the following criteria: age > 60 years, temperature > 38.3 °C, albumin level < 2.5 mg/dL, peripheral WBC count > 15,000 cells/mm^3^, endoscopic proof of pseudomembranous colitis, and intensive care unit admission [12]. The ATLAS scoring system has been considered useful for predicting treatment response and for the categorization of patients in CDI therapeutic studies. It considers the following criteria: age, systemic antibiotic treatment, leukocyte count, serum albumin, and serum creatinine as a measure of renal function [13].

Regarding financial cost, CDI is one of the most expensive nosocomial infections [14,15]: in the US, the mean CDI-associated excess total healthcare costs have been estimated at $13,476 [16], in Europe €15,242 [17], in Italy €10,224, with CDI-associated length of stay (LOS) serving as the primary cost driver [18].

Also, the high rate of CDI recurrences (rCDIs) results in increased treatment costs [17].

Up to 30% of patients with CDI who were successfully treated experience rCDI. Treatment with PPIs, healthcare facility-associated CDI, and a prior episode of rCDI were identified as additional risk factors for s/rCDI [11].

Our study aims to analyze CDI incidence in the last ten years at the Palermo University Hospital, ascertain which departments were most involved, evaluate the treatments, determine the attributable mortality, determine the incidence of rCDI, and search for risk factors for mortality and rCDI. Lastly, the study checked whether the CDI was coded accurately on the hospital standard discharge forms (H-SDF [19,20]).

## 2. Materials and Methods

Raw data were obtained by the routine activity of the Antimicrobial Stewardship Team of AOU Policlinico “P. Giaccone” regarding the monitoring of nosocomial infections and prescribing appropriateness. Our analysis was conducted using anonymized data collected from all H-SDF between January 2013 and June 2022 in the tertiary care University Hospital Policlinico “P. Giaccone” in Palermo (Italy). H-SDF is filled at the patient’s discharge by the Medical Doctor. In addition to the anagraphic information, it contains up to six ICD-9-CM-coded diagnoses and five procedures [20]. The ICD-9 code 00845, which denotes CDI with or without complications, was used to identify CDI cases. Uncoded CDI cases were analyzed using an anonymized dataset generated by the Microbiology Unit from the collection of all conducted microbiological tests. In the study period in our Polyclinic, only diarrheic samples were tested for *C. difficile*, and neither routine screening for *C. difficile* colonization nor a test of cure was done.

Cases were classified as hospital-acquired (HA) if CDI developed after 48 h from admission; community-acquired (CA) if CDI developed in the first 48 h from admission. Demographic data, the Charlson Comorbidity Index (CCI), clinical and laboratory characteristics, type of diagnosis, previous infection, in-hospital PPI and antibiotic use and duration in the four weeks preceding CDI onset, CDI therapy, rCDI rate, incidence, distribution per ward, mortality, and H-SDF coding rate were considered. To establish a link between CDI and death, CDI death was reviewed and discussed separately by two authors. Death was assessed only considering the in-hospital mortality. The deceased cases were subdivided into three main categories, as already done by Hota et al. [21]: CDI directly caused or strongly contributed to the death; CDI somewhat contributed to the death or was unrelated to the death; and the information was insufficient to determine the role of CDI in the death.

All the definitions of CDI, rCDI, and sCDI, plus the mode of diagnosis, were in accordance with ESCMID-2021-guidelines [9]; in detail, an episode of CDI is defined as clinical findings compatible with CDI and microbiological evidence of *C. difficile*-free toxins by enzyme immunoassay or a positive nucleic acid amplification test (NAAT) without reasonable evidence of another cause of diarrhea. An rCDI is defined as CDI that recurs within eight weeks after a previous episode, provided the symptoms of the prior episode are resolved after the completion of initial treatment.

This study was approved by the Ethics Committee “Palermo I”, Palermo, Italy (Verbal n.1 25/0172023).

## 3. Statistical Analysis

The data were analyzed using IBM SPSS Statistics. To describe the demographic and clinical characteristics of the study population, categorical variables were summarized with frequencies and percentages, and continuous variables with median and IQR (interquartile range) or mean and SD (standard deviation).

The 95% confidence interval (CI) was calculated for continuous data. Contingency tables were analyzed by the two-tailed Χ^2^ test or Fisher’s exact test, and continuous data using the Student’s t-test. The Pearson correlation coefficient was computed to verify the existence of correlations between variables.

The length of time between being admitted to the hospital and being diagnosed with CDI and dying was examined using the Kaplan−Maier method. Different curves were analyzed using the Logrank test.

A 2-sided *p*-value < 0.05 was considered significant for all analyses. A multivariable analysis was performed to identify risk factors for death and rCDI. A propensity score matching was computed to reduce bias due to confounding variables to estimate the likelihood of rCDI based on treatment with vancomycin or fidaxomicin.

A multivariable analysis including significant variables associated with mortality by univariate analysis was performed. In order to optimize the performance of the multivariable analysis the number of variables to be included was reduced by replacing white blood cell count, serum albumin, and serum creatinine with the ATLAS score. It was not considered useful to include in the multivariable analysis septic shock because it is obviously linked to mortality.

ROC curve was used to calculate continuous variable threshold values.

## 4. Results

**Figure 1:**
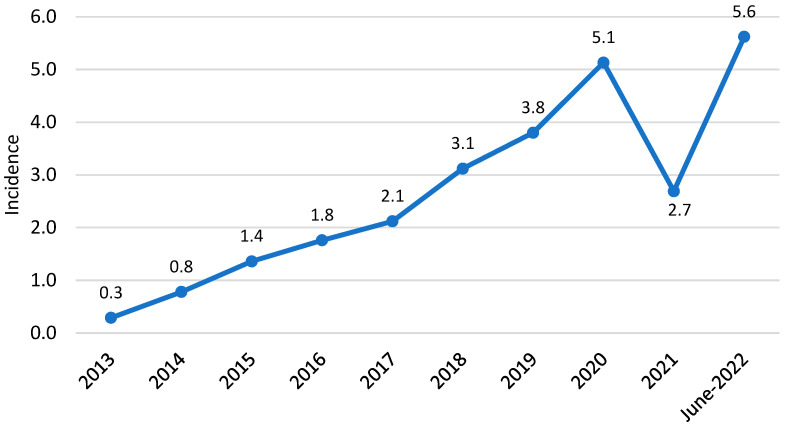
CDI incidence per 10,000 patient days.

**Figure 2:**
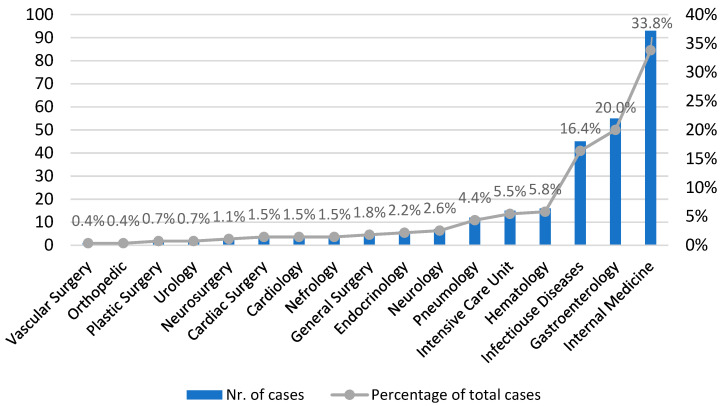
CDI cases by hospital wards.

During the study period, 275 CDI cases were diagnosed. Incidence was 0.3 per 10.000 PD in 2013 and progressively increased to 5.6 in the first semester of 2022 for an 18.7-fold increase (see Figure 1). Medicine, surgery, and the intensive care unit recorded 88%, 6.5%, and 5.4% of cases, respectively. Figure 2 shows that 33.8%, 20%, and 16.3% of all patients were seen in the Internal Medicine, Gastroenterology, and Infectious Disease (ID) Units, respectively.

**Figure 3:**
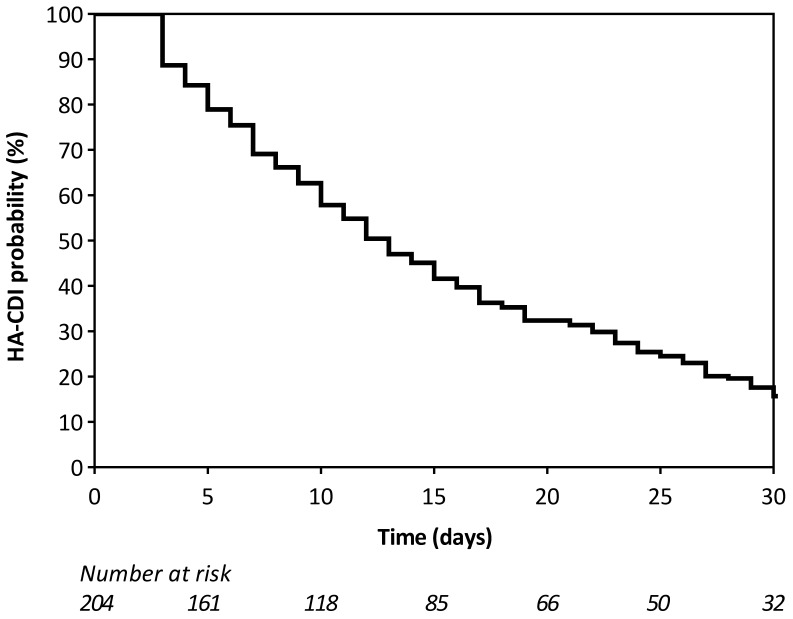
Time elapsed between hospital admission and CDI onset for HA-CDI cases.

The characteristics of *C. difficile*-infected patients are reported in Table 1: 75% were HA, and 25% were CA. For HA-CDI, the median time between admission and CDI onset was 13 days (IQR 7–25) (see Figure 3).

A total of 67% of our patients had a history of infections: 37.6% had pneumonia, 32.6% had urinary tract infections, and 31.5% had bloodstream infections (of these, 57.7%, 36.5%, and 7.7% were caused by gram-positive and gram-negative bacteria, and *Candida* spp., respectively).

A total of 9 episodes of candidemia were diagnosed immediately preceding and succeeding CDI diagnosis, with an overall rate of 3.3%: 4 cases before and 5 after CDI.

CDI was diagnosed in 65.8% of cases by combining 2 diagnostic methods: EIA for GDH and EIA for toxins, or NAAT (see Supplementary Materials Table S1). Toxins involved were B and A, respectively, in 74% and 45% of cases (differently combined), and binary toxin was in 4%.

**Figure 4:**
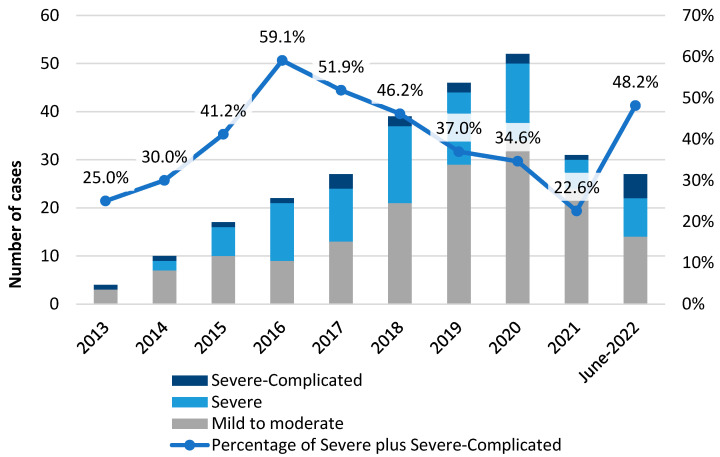
CDI Severity cases according to ESCMID criteria along the study decade.

A total of 40.4% of CDI were severe/severe-complicated, and 31.4% had a Zar score ≥ 2. The rate of severe/severe-complicated cases progressively increased over the decade 1.9 times from 25 to 48% (see Figure 4, and Supplementary Materials Table S2). In 17% of cases overall and 24.7% of the cases diagnosed since 2019, fidaxomicin 200 mg q12h was given (the year in which the drug was available in our hospital). Fidaxomicin 200 mg q12h was administered, respectively, in 17% of cases overall and in 24.7% of the cases diagnosed since 2019 (the year in which the drug was available in our hospital). A switch to a more effective option was required in 23.5% of cases, and adjunctive treatment (IV tigecycline and rectal vancomycin) in one case. One patient underwent a colectomy. Intensive care was necessary in 2.5% of cases (see Supplementary Materials Table S3). Fidaxomicin was the most prescribed for CDI cases with ≥3 risk factors of a negative outcome (see Supplementary Materials Table S4). A total of 4 patients received no specific treatment: for 1 of them, the sole antibiotic treatment interruption was sufficient for clinical resolution, while 3 of them died after 7, 9, and 10 days, respectively, from diagnosis.

**Figure 5:**
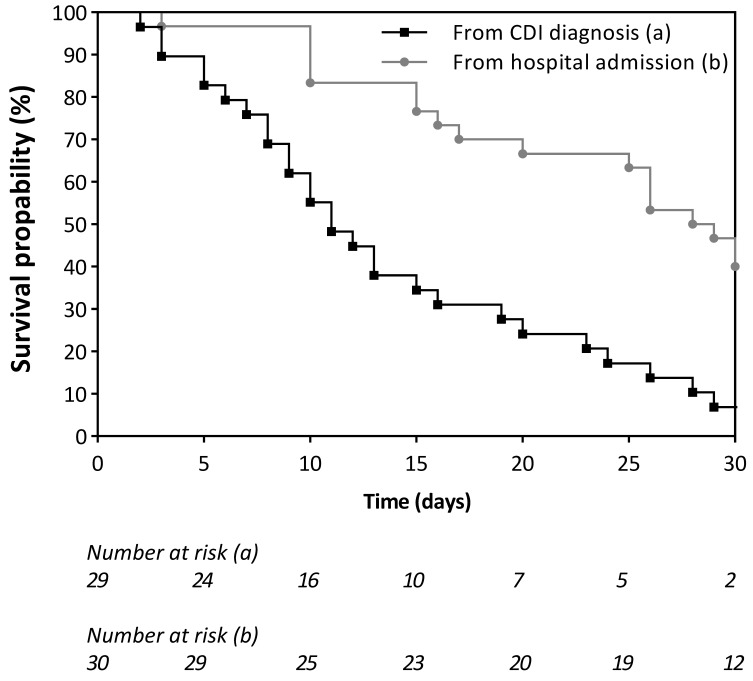
Survival curves: Time elapsed for CDI deceased cases between CDI onset and death and between hospitalization and death.

A total of 31/275 (11.3%) of CDI cases died, with a 30-day mortality of 10.5% (29/275). Some 41.9% (13/31) of deaths were directly attributable to *C. difficile*, or it strongly contributed to death; 35.5% (11/31) were unrelated to CDI; in 22.6% (7/31) of the cases, data were not adequate to establish the clinical correlation. CDI-attributable mortality was 4.7% (13/275). The median time between CDI diagnosis and death was 11 days (IQR 6–25), and the median time between hospital admission and death was 28 days (IQR 15–50) (see Figure 5).

CCI, hemodialysis, previous infection, higher white blood cell count and creatinine, lower albumin, and vomit at presentation, higher creatinine increase from baseline, and higher Zar score were all associated with the occurrence of death in the univariate analysis (see Table 1).

Only hemodialysis was linked to an increased risk of death, according to the multivariable analysis (see Table 1). In detail, 50% of patients with HD died, compared to 8.8% of those without HD (aOR 8; CI 2–32.5).

There were 11 (4%) cases of rCDI all observed between 2019 and 2022; the mean age was 69.2 (range 33–88), the sex ratio (M:F) was 1:1.75, the median LOS 41 days (IQR 38), and the median time between the first episode and rCDI diagnosis 22.5 days (IQR 16.2). The 36.4% of rCDI were severe or severe-complicated cases, 55% received fidaxomicin, and 64% bezlotoxumab. Only one patient died. None of the factors investigated was linked to an increased risk of developing an rCDI in either a univariate or multivariable analysis (see Table 1).

The likelihood of rCDI in patients treated with fidaxomicin was lower in comparison to the one in patients treated with vancomycin, and it was computed by a propensity score that yielded a high, but not statistically significant odds ratio (OR 2.35, 95% CI 0.42–13.14; *p*-value 0.33).

**Figure 6:**
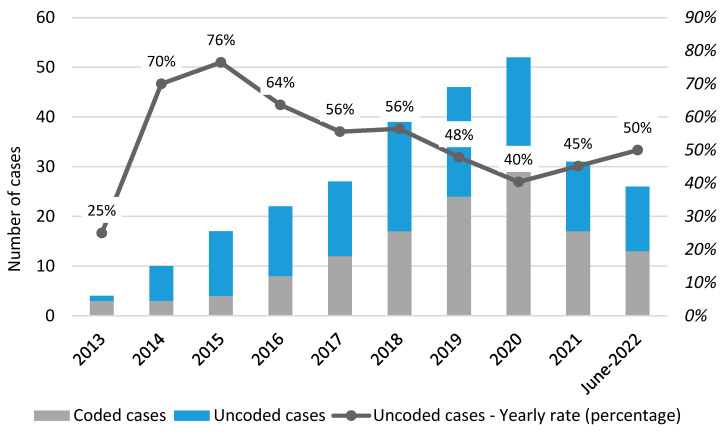
CDI coding rate over study decade.

During the 10 years, only 48.1% of CDI cases were coded in H-SDF. The percentage of missing H-SDF coding decreased from 70–76% to 50% during the decade (see Figure 6). Cardiology, medicine, and ID wards had the highest CDI coding rates, with respective rates of 75%, 66.6%, and 62.2% (see Supplementary Materials-Figure S1).

## 5. Discussion

In our analysis, the incidence of CDI was 0.3 and 5.6 per 10,000 PDs in 2013 and 2022, respectively (18.7-fold increase), which is much lower than the PPS-EUCLID’s predicted incidence of 9.5 and 7.2 per 10,000 PDs in 2011–2012 and 2012–2013, respectively [6]. This disparity is most likely due to a lack of awareness of CDI, particularly in surgical and intensive care units, where only 6.5 and 5.4% of cases were identified in our study (compared to 18 and 10% reported by ECDC) [22]. Indeed, EUCLID had previously estimated that 25% of CDI cases in Europe lacked a diagnosis; an Italian study documented CDI underdiagnosis at 11% [23]. So, the lower incidence described in our hospital may be due to underdiagnosis.

It is not easy to explain the sharp decrease in incidence observed in 2021 as well as the increase in sCDI cases observed in 2022. These two phenomena may have a multifactorial origin, most likely related to the COVID-19 pandemic. Indeed, as already documented in previous studies, the decrease in CDI incidence during the COVID-19 pandemic could be due to improved hygiene, more protective clothing, social distancing, and fewer inpatients [24,25,26,27,28,29]. As has already been hypothesized in other studies [24], the higher percentage of sCDI during the second COVID-19 wave may be related to delayed CDI diagnosis due to limited hospital referrals.

There is a paucity of data on CDI incidence and clinical manifestations in COVID-19 patients. Indeed, we cannot exclude that the decrease in CDI incidence observed in 2021 could partially depend on a misdiagnosis due to an erroneous attribution of the diarrheal symptoms to COVID-19 rather than to *C. difficile*.

Our sCDI rate was 40.4% (like other Italian data [30]), and ECDC previously reported a rate of 16.8% [2]. Data on sCDI are not easily comparable because the severity definition has changed. In any case, we observed a progressive increase in severity (from 25 to 48.1%), which Esteban-Vasallo et al. [31] previously described.

Some 75% of the 275 CDI cases were classified as HA and 25% as CA. In 2016, the ECDC-funded coordination of CDI surveillance [2] discovered a comparable rate of CA CDI (25.4%), though a higher rate (45.55%) was recently observed in Italy [30]. In our study, the median time between admission and the onset of CDI was comparable to the ECDC (13 vs. 9–11 days) [22].

Our LOS was 22 days, which was significantly longer than the CDC’s 8-day estimate [32] but comparable to other multicentric European [17] and Italian studies [18,30].

The most notable findings in terms of preceding and concurrent infection concern candidemia, which was documented in 9 instances (3.2%), with 55.6% of episodes occurring after CDI and a mean delay of 15.5 days between candidemia and CDI (IQR 4.3–30.5). This is consistent with the literature, which shows a close relationship between candidemia and CDI due to their similar pathogenic mechanisms [33], with candidemia occurring 66% of the time after CDI [34].

All-cause and attributable mortality in our study were 11.3 and 4.7% vs. 20.7% and 3.9 reported by ECDC [2], even if a recent multicentric European study reported a mortality rate of 13% [17].

In our multivariable analysis, only hemodialysis predicted a higher mortality risk (aOR 8; CI 2–32.5).

As a result, caution must be exercised in these patients to avoid incorrect or excessively long-lasting antibiotic treatments.

There is not much data regarding the occurrence of CDI among HD patients. Nonetheless, they appear to have a 2-fold higher risk of developing CDI and a 2-fold higher fatality rate than the general population, with rates ranging from 13 to 69% [35,36,37,38,39,40,41,42]. It could be due to frequent antimicrobial exposure, frequent hospitalizations, and significant immune dysfunction in HD patients [43].

In comparison to our 4% of rCDI, higher rates of rCDI were documented by the ECDC (7.9% in 2016) [2], in Canada and South America (12 and 40%) [44,45], and in another Italian study (21%) [30]. While our study was not intended to detect rCDI, we cannot rule out the possibility that some cases occurred after discharge, at home, or in other hospitals or healthcare facilities.

A multivariable study revealed no risk factors that predicted the risk of rCDI, possibly due to their limited number. Even if there was no statistically significant difference in the number of rCDI between patients taking vancomycin or fidaxomicin, more frail patients were administered fidaxomicin.

In terms of treatment, the most notable result was the use of fidaxomicin in our hospital, which, despite being available only since 2019, was used much more widely than in other Italian centers [30]. Currently, there are no ECDC data regarding the utilization of fidaxomicin.

Despite its high cost, which currently prevents widespread use, fidaxomicin is not only comparable to vancomycin in terms of clinical cure, but also superior in terms of fewer rCDI, faster symptom resolution, and higher sustained response rates. Indeed, the benefit has been demonstrated to be higher in most fragile patients [46,47]. Risk group stratification strategies are needed to identify patients who are most likely to benefit from fidaxomicin [48].

In our population, bezlotoxumab was used in 64% of rCDI (7 patients). Recent data demonstrate a marked efficacy of bezlotoxumab in combination with standard of care in the prevention of rCDI and death [49]. Data also suggests that the benefit might be even greater in patients older than 70 years and in those treated with vancomycin as the standard of care [49].

The treatment of severe-complicated CDI cases is still being debated. The ESCMID guidelines take into account combination therapy with adjunctive IV tigecycline. Using IV metronidazole is discouraged, but this latter indication appears to be lacking in evidence [50].

Other European studies, conducted in countries lacking a surveillance system, were based on H-SDF analysis [31,51,52]. In the absence of microbiology databases, H-SDF is the only tool available to track the occurrence of CDI. CDI diagnoses, however, are not always coded in H-SDF. Indeed, the cross-check between H-SDF and the Microbiology Database in our study revealed a rate of 51.9% undercoding, with a decreasing trend over the decade. As a result, this is still the only tool always available to track CDI incidence in every hospital, regardless of its level, given that Microbiology datasets are not routinely filled in all hospitals while H-SDF is.

## 6. Conclusions

Our study confirms the CDI incidence increase already documented worldwide. Death rates were higher in those who were receiving hemodialysis or showed symptoms of sCDI. Maximum attention should be paid to preventing people on hemodialysis from getting CDI.

We call for the implementation of CDI surveillance and strongly recommend always coding the CDI diagnosis in H-SDF to help track infection rates.

## Figures and Tables

**Table 1:** Demographic, clinical, and laboratory characteristics, previous infections of the total population and of those alive and dead, with univariate and multivariable analysis.

Outcome Clinical									
Variables	All (n:275)	Alive (n:244)	Dead (n:31)	Crude Or (CI 95%)	AOR (CI 95%)	*p*-Value	No Recurrence (n:264)	Recurrence (n:11)	*p*-Value
Epidemiological characteristics									
Male	120 (43.6%)	108 (44.3%)	12 (38.7%)	1			116 (43.9%)	4 (36.4%)	
Female	155 (56.4%)	136 (55.7%)	19 (61.3%)	1.3 (0.6–2.7)			148 (56.1%)	7 (63.6%)	
Age (average ± STD) <57 ≥57	65.8 ± 18.4 73 (26.5%) 202 (73.5%)	65.2 ± 19.1 69 (28.3%) 175 (71.7%)	71.2 ± 10.7 4 (12.9%) 27 (87.1%)	1 2.7 (0.90–7.9)			65.7 ± 18.4 72 (27.2%) 192 (72.7%)	69.1 ± 16.9 1 (9.1%) 10 (90.9%)	
Nationality									
Italian Not Italian	265 (96.4%) 10 (3.6%)	235 (96.3%) 9 (3.7%)	30 (96.8%) 1 (3.2%)	1 0.9 (0.1–7.1)			256 (97.0%) 8 (3.0%)	9 (81.8%) 2 (18.2%)	0.228
Outcome									
LOS median (IQR)	22 (12–39)	21 (12–39)	28 (15–50)				22 (12–38)	41 (18–56	
<25 ≥25	149 (54.2%) 126 (45.8%)	138 (56.6%) 106 (43.4%)	11 (35.5%) 20 (64.5%)	1 2.4 (1.1–5.1)	1 2.7 (1–7.4)	0.057	146 (55.4%) 118 (44.7%)	3 (27.2%) 8 (72%)	0.076
Recurrence No Yes	264 (96.0%) 11 (4.0%)	264 (95.9%) 10 (4.1%)	30 (96.8%) 1 (3.2%)	1 0.8 (0.1–6.3)			-	-	
Risk factors and comorbidities									
CCI (average ± STD) <5 ≥5	5.3 ± 2.8 91 (33.1%) 184 (66.9%)	5.1 ± 2.9 88 (36.1%) 156 (63.9%)	6.3 ± 1.7 3 (9.7%) 28 (90.3%)	1 5.3 (1.6–17.8)			5 ± 3 90 (34%) 176 (66%)	7 ± 4 3 (27%) 8 (72.7%)	
Chronic kidney disease (n:275)							203 (76%) 61 (23.1%)	7 (63.6%) 4 (36.4%)	
No Yes	210 (76.4%) 65 (23.6%)	187 (76.6%) 57 (23.4%)	23 (74.2%) 8 (25.8%)	1 1.1 (0.5–2.7)					
Hemodialysis (n:275)									
No Yes	259 (94.2%) 16 (5.8%)	236 (96.7%) 8 (3.3%)	23 (74.2%) 8 (25.8%)	1 10.3(3.5–29.9)	1 7.6 (2–27.3)	**0.001**	249 (94.3%) 15 (5.7%)	10 (90.1%) 1 (9.1%)	
Diabetes mellitus II (n:275)							211 (79.9%) 53 (20.1%)	8 (72.7%) 3 (27.3%)	
No Yes	219 (79.6%) 56 (20.4%)	195 (79.9%) 49 (20.1%)	24 (77.4%) 7 (22.6%)	1 1.2 (0.5–2.8)					
Chronic liver disease (n:275)									
No Yes	236 (85.8%) 39 (14.2%)	209 (85.7%) 35 (14.3%)	27 (87.1%) 4 (12.9%)	1 0.9 (0.3–2.7)			226 (85.6%) 38 (14.4%)	10 (90.1%) 1 (9.1%)	
Immunosuppression (n:275)							230 (87.1%) 34 (12.9%)	10 (90.1%) 1 (9.1%)	
No Yes	240 (87.3%) 35 (12.7%)	215 (88.1%) 29 (11.9%)	25 (80.6%) 6 (19.4%)	1 1.8 (0.7–4.7)					
Inflammatory bowel disease (n:275)									
No Yes	240 (87.3%) 35 (12.7%)	210 (86.1%) 34 (13.9%)	30 (96.8%) 1 (3.2%)	1 0.2 (0–1.6)			230 (87.1%) 34 (12.9%)	10 (90.1%) 1 (9.1%)	
Previous infections (n:266)									
No Yes	88 (33.1%) 178 (66.9%)	83 (35.3%) 152 (64.7%)	5 (16.1%) 26 (83.9%)	1 2.9 (1–7.7)	1 0.7 (0.2–2.4)	0.539	87 (34.1%) 168 (65.9%)	1 (9.1%) 10 (90.1%)	
Use of antibiotics (n:138)									
No Yes	14 (10.1%) 124 (89.9%)	10 (9.3%) 97 (90.7%)	4 (12.9%) 27 (87.1%)	1 0.7 (0.2–2.4)			12 (9.4%) 115 (90.6%)	2 (18.1%) 9 (81.8%)	
Use of PPI (n:111)							24 (25.5%) 76 (74.5%)	5 (45.5%) 6 (54.5%)	
No Yes	29 (26.1%) 82 (73.9%)	21 (22.1%) 74 (77.9%)	8 (50%) 8 (50%)	1 0.3 (0.1–0.8)					
Clinical features									
Diarrhea (n:136)									
No Yes	7 (5.1%) 129 (94.9%)	5 (4.4%) 108 (95.6%)	2 (8.7%) 21 (91.3%)	1 0.5 (0.1–2.7)			7 (5.6%) 118 (94.4%)	0 (0%) 11 (100%)	
Abdominal pain (n:135)									
No Yes	92 (68.1%) 43 (31.9%)	76 (67.9%) 36 (32.1%)	16 (69.6%) 7 (30.4%)	1 0.9 (0.3–2.4)			84 (67.7%) 40 (32.3%)	8 (72.7%) 3 (27.3%)	
Fever (n:275)							168 (63.3%) 96 (36.4%)	11 (100%) 0 (0%)	0.996
No Yes	179 (65.1%) 96 (34.9%)	163 (66.8%) 81 (33.2%)	16 (51.6%) 15 (48.4%)	1 1.9 (0.9–4)					
Vomit (n:135)									
No Yes	124 (91.9%) 11 (8.1%)	106 (94.6%) 6 (5.4%)	18 (78.3%) 5 (21.7%)	1 4.9 (1.3–17.8)			113 (91.8%) 11 (8.9%)	11 (100%) 0 (0%)	
Ileum (n: 135)							120 (96%) 5 (4.0%)	10 (100%) 0 (0%)	
No Yes	130 (96.3%) 5 (3.7%)	108 (96.4%) 4 (3.6%)	22 (95.7%) 1 (4.3%)	1 1.2 (0.1–11.5)					
Pseudomembranes (n:275)									
No Yes	268 (97.5%) 7 (2.5%)	237 (97.1%) 7 (2.9%)	31 (100%) 0 (0%)	1 -			258 (97.7%) 6 (2.3%)	10 (90.9%) 1 (9.1%)	
Shock (n:275)							251 (95.1%) 13 (4.9%)	11 (100%) 0 (0%)	
No Yes	262 (95.3%) 13 (4.7%)	241 (98.8%) 3 (1.2%)	21 (67.7%) 10 (32.3%)	1 38.2 (9.8–149.8)					
Intensive care unit stay (n:273)									
No Yes	266 (97.4%) 7 (2.6%)	242 (99.6%) 1 (0.4%)	24 (80.0%) 6 (20.0%)	1 60.5 (10–523.6)			256 (97.7%) 6 (2.3%)	10 (90.9%) 1 (9.1%)	
Other infections—Candidemia (n:270)									
No Yes	266 (98.5%) 4 (1.5%)	237 (98.3%) 4 (1.7%)	29 (100%) 0 (0%)	1 -			255 (98.5%) 4 (1.5%)	11 (100%) 0 (0%)	
Severity according to Zar score (n:242)									
<2 ≥2	166 (68.6%) 76 (31.4%)	155 (72.8%) 58 (27.2%)	11 (37.9%) 18 (62.1%)	1 4.4 (1.9–9.8)			160 (69.2%) 71 (30.3%)	6 (54.5%) 5 (45.4%)	
Severity according to ESCMID 2021 definition (n:275)									
Not severe Severe/complicated	164 (59.6%) 111 (40.4%)	159 (65.2%) 85 (34.8%)	5 (16.1%) 26 (83.9%)	1 9.7 (3.6–26.2)			157 (59.5%) 107 (40.5%)	7 (63.6%) 4 (36.4%)	
ATLAS score (n:130)									
≤5 >5	105 (80.8%) 25 (19.2%)	84 (83.2%) 17 (16.7%)	21 (72.4%) 8 (27.6%)	1 1.9 (0.7–4.9)	1 1.6 (0.5–4.6)	0.406	96 (80.7%) 23 (19.3%)	9 (81.8%) 2 (18.2%)	
Laboratory tests									
White blood cells (n:275)									
Average ± STD <12,880 ≥12,880	10,9 ± 10.4 191 (69.5%) 84 (30.5%)	9.9 ± 7.6 177 (72.5%) 67 (27.5%)	18.5 ± 21 14 (45.2%) 17 (54.8%)	1 3.2 (1.5–6.9)			10,9 ± 10,5 182 (69%) 82 (31%)	10,5 ± 5,0 9 (81.8%) 2 (18.2%)	
Basal creatinine (n:273)									
Average ± STD <0.96 ≥0.96	1.19 ± 1.0 154 (56.4%) 119 (43.6%)	1.1 ± 1.0 143 (59.1%) 99 (40.9%)	1.5 ± 1.4 11 (35.5%) 20 (64.5%)	1 2.6 (1.2–5.7)			1.1 ± 1.0 148 (56.4%) 114 (46.6%)	1.2 ± 1.0 6 (54.5%) 5 (45.5%)	
Creatinine during infection (n: 273)									
Average ± STD <1.87 ≥1.87	1.3 ± 1.2 233 (85.3%) 40 (14.7%)	1.2 ± 1.1 214 (88.4%) 28 (11.6%)	1.9 ± 1.7 19 (61.3%) 12 (38.7%)	1 4.8 (2.1–11.1)			1.3 ± 1.2 225 (88.9%) 37 (14.1%)	1.1 ± 0.7 8 (72.7%) 3 (27.2%)	
Increase (%) in creatinine from baseline (n:273)									
Average ± STD <42% ≥42%	14.1 ± 51.4 137 (50.4%) 135 (49.6%)	10.3 ± 37 126 (52.3%) 115 (47.7%)	43.5 ± 109.2 11 (35.5%) 20 (64.5%)	1 2 (0.9–4.3)			14.1 ± 51.7 127 (48.6%) 134 (51.4%)	12.0 ± 45.4 10 (90.9%) 1 (9.1%)	
Albumin during infection (n:243)							3.0 ± 0.64 141 (60.8%) 91 (39.2%)	2.7 ± 0.4 3 (27.3%) 8 (72.7%)	
Average ± STD >2.80 ≤2.80	3 ± 0.6 144 (59.3%) 99 (40.7%)	3 ± 0.64 133 (62.1%) 81 (37.9%)	2.71 ± 0.53 11 (37.9%) 18 (62.1%)	1 2.7 (1.2–6)					
Antibiotic therapy for CDI (n:136)									
Fidaxomicin Metronidazole Vancomycin	23 (17%) 15 (11%) 98 (72%)	10 (10.1%) 13 (13.1%) 76 (76.8%)	13 (35.2%) 2 (5.4%) 22 (59.4%)	0.08 (0.0–0.2) 0.07 (0.0–0.2) 0.09 (0.0–0.1)			21 (16.8%) 15 (12%) 89 (71.2%)	2 (18.2%) 0 (0%) 9 (81.8%)	

LOS: length of stay; CCI: Charlson Comorbidity Index; PPI: proton pump inhibitors; OR: odds ratio; AOR: adjusted odds ratio; CI: confidence interval.

## Data Availability

Dataset available if required.

## Supplementary Materials

The following supporting information can be downloaded at: https://www.mdpi.com/article/10.3390/antibiotics12050837/s1, Table S1: Modality of CDI diagnosis; Table S2: Number of CDI cases per severity over the years and number of dead and recurrent CDI per year; Table S3: CDI treatment in the global population and in CDI cases diagnosed since 2019; Table S4: CDI treatment distribution according to the number of risk factors for death in CD infected patients; Figure S1: CDI Coding rate.
