# Supplementary material for: Clostridioides difficile Infection in an Italian Tertiary Care University Hospital: A Retrospective Analysis
Source: Antibiotics (Basel). 2023 Apr 30;12(5):837. doi: 10.3390/antibiotics12050837 (PMC10215700; doi:10.3390/antibiotics12050837)
Supplement: Supplementary file 1 [file antibiotics-12-00837-s001.zip › antibiotics-2252129-supplementary.pdf]

	2 – Step Diagnosis	1 – Method Diagnosis	Total
**GDH + EIA Toxin**	47.6% (132/275)		
**GDH + NAAT**	17.8% (49/275)		
			65.8% (181/275)
**GDH**		21.8% (60/275)	
**EIA Toxin**		9.4% (26/275)	
**NAAT**		2.9% (8/275)	
			34.1% (94/275)

Table S1 - Modality of CDI diagnosis. All data are expressed as percentages (number of cases/total population).

	Mild-to-moderate CDI	Severe CDI	Severe-complicated CDI	Total	Death	Recurrence
2013	3	0	1	4	1	
2014	7	2	1	10	2	
2015	10	6	1	17	2	
2016	9	12	1	22	3	
2017	13	11	3	27	2	
2018	21	16	2	39	6	
2019	29	15	2	46	3	3
2020	34	16	2	52	5	1
2021	24	6	1	31	2	4
June-2022	14	8	5	27	5	3
Total	164	92	19	275	31	11

Table S2 - Number of CDI cases per severity over the years and number of dead and recurrent CDI per year.

	Global	From 2019
Metronidazol	11% (15/136)	3.2% (3/93)
Vancomycin	72% (98/136)	72% (67/93)
Fidaxomycin	17% (23/136)	24.8% (23/93)
Adjunctive treatment	1.5% (2/136)	2.1% (2/93)
Colectomy	0.36% (1)	0%
ICU requirement	2.5% (7/273)	2.6% (4/156)
Switch to vancomycin	75% (24/32)	53% (9/17)
Switch to fidaxomycin	25% (8/32)	47% (8/17)

Table S3 - CDI treatment in the global population and in CDI cases diagnosed since 2019. All data are expressed as percentages (number of cases/considered population).

Demographics	Vancomycin (n=98) ≤ 2 RF n = 25	Vancomycin (n=98) ≥3 RF n = 73	Fidaxomicin (n=23) ≤ 2 RF n = 3	Fidaxomicin (n=23) ≥3 RF n = 20	Metronidazole (n=15) ≤ 2 RF n = 5	Metronidazole (n=15) ≥3 RF n = 10
Age Mean (SD)	53.36 (17.85)	73.90 (12.23)	49.67 (31.07)	73.70 (11.40)	57.40 (14.78)	79.60 (9.05)
> 65 y (%)	6 (25.0%)	66 (91.0%)	1 (33.3%)	16 (80.0%)	2 (60.0%)	10 (100%)
Immunocompromised (%)	2 (7.1%)	9 (12.8%)	0 (0%)	5 (25.0%)	2 (40.0%)	3 (30.0%)
Severe CDI* (%)	2 (8.0%)	40 (54.7%)	1 (33.3%)	8 (44.4%)	0 (0%)	7 (70.0%)
ICU (%)	0 (0%)	6 (7.8%)	0 (0%)	1 (5.0%)	0 (0%)	1 (10.0%)
Previous antibiotic therapy (%)	21 (83.3%)	71 (97.4%)	2 (66.6%)	20 (100%)	2 (40.0%)	10 (100%)
Pseudomembranous (%)	0 (0%)	3 (3.8%)	3 (100%)	20 (100%)	0 (0%)	1 (10.0%)
**Risk factors**						
0	0 (0%)		0 (0%)		1 (6.7%)	
1	7 (7.5%)		0 (0%)		1 (6.7%)	
2	19 (18.9%)		3 (13.0%)		3 (20.0%)	
3	40 (40.6%)		11 (47.8%)		2 (13.3%)	
4	25 (25.5%)		8 (34.8%)		5 (33.3%)	
5	7 (7.5%)		1 (4.3%)		3 (20.0%)	

RF: risk factors, y: years, *Zar score ≥2, CDI: *Clostridium difficile infection*, ICU: intensive care unit.

Table S4 - CDI treatment distribution according to the number of risk factors for death in CD infected patients.

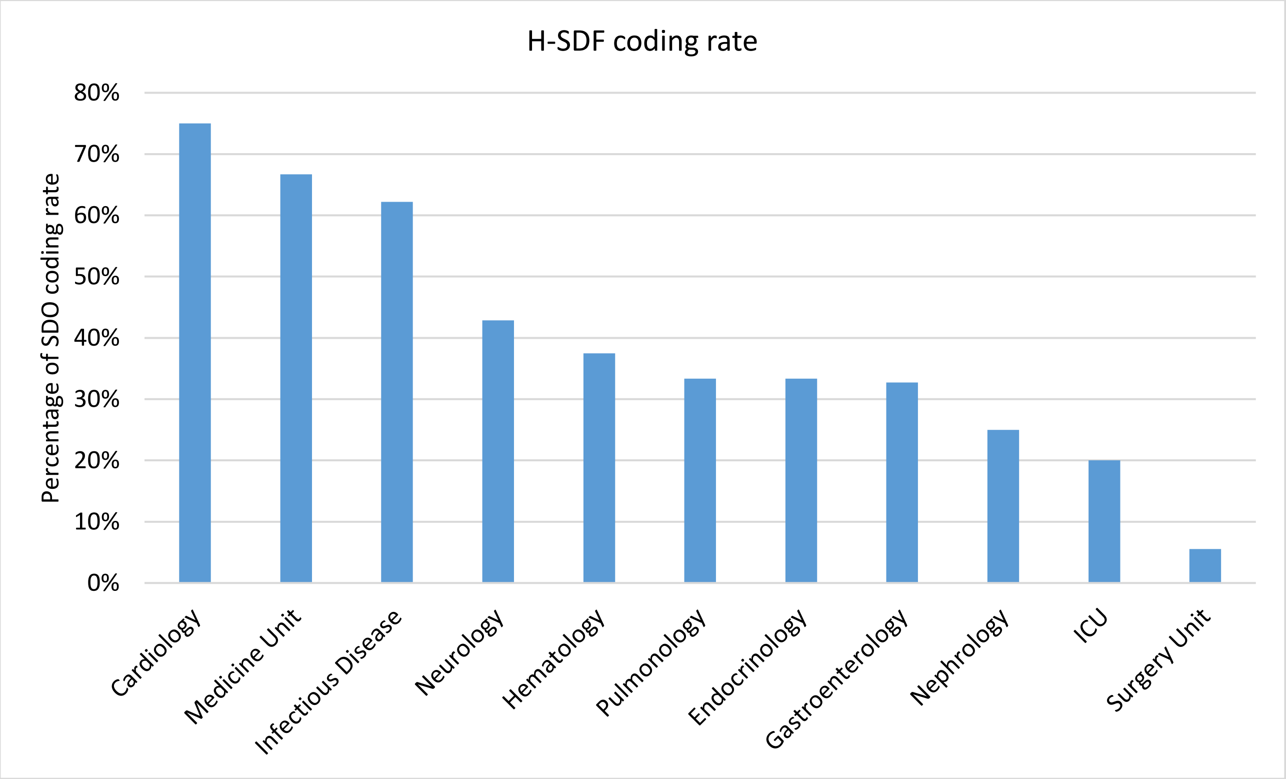

Figure S1 - CDI Coding rate for each ward.
